# LiDAR-Based Safety Envelope Detection with Accelerometer and DTW for Intrusion Localization in Roller Coasters

**DOI:** 10.3390/mi16091062

**Published:** 2025-09-19

**Authors:** Huajie Wang, Zhao Zhao, Yifeng Sun, Weikei Song

**Affiliations:** 1 Technology Innovation Center of Health Management of Large-Scale Amusement Device, State Administration for Market Regulation, Beijing 100029, China; 2 China Special Equipment Inspection and Research Institute, Beijing 100029, China

**Keywords:** roller coaster, safety envelope, LiDAR, dynamic time warping

## Abstract

Autonomous vehicles, submersible robotic systems and drones, and other human-carrying equipment consistently adhere to a safety perimeter, ensuring collision-free navigation amidst surrounding objects. In contrast, roller coaster vehicles, despite being constrained to a predetermined track, necessitate frequent safety distance detection owing to the variability introduced by trees and decorative installations. Passengers’ limbs may protrude beyond vehicle boundaries, posing a collision hazard. The motion range of limbs, influenced by vehicle-specific conditions, mismatches standardized safety volumes (cylindrical, cubic, and rectangular) designed for mobile entities. The roller coaster industry’s current practice involves a moving safety frame, which visually inspects for collisions to assess safety distances, which is cumbersome and prone to oversight in intricate settings. Therefore, this study introduces a novel safety envelope detector (SE-detector). It creates a customer-defined virtual safety envelope around the roller coaster vehicle and measures the safety distance based on LiDAR (Light Detection and Ranging) to detect the intrusions of obstacles. Meanwhile, this SE-detector also innovatively integrated an accelerometer to synchronously measure the acceleration of the vehicle. The measured acceleration will be aligned with simulated sequences by dynamic time warping (DTW) algorithms to pinpoint intrusion location. Additionally, a wide-angle camera is also deployed to enhance perception of the surrounding environment. The SE-detector developed in this study has the capability to record inspection results. It is expected to enhance the inspection capabilities of the safety envelope for roller coasters, thereby improving the efficiency of safety distance inspection.

## 1. Introduction

Safety envelope research has a broad research foundation spanning various industries. For instance, in the field of autonomous navigation and assisted driving, ensuring collision avoidance necessitates the establishment of a virtual safety distance matrix that encapsulates the core structures of these moving entities [1,2]. These systems continuously assess distances using advanced technologies such as LIDAR, visual imaging modalities, and acoustic sonar, thereby enabling obstacle detection and avoidance. Nevertheless, prior research has frequently oversimplified the geometries of safety envelopes, typically adopting cylindrical or rectangular configurations [3,4,5], overlooking the complex range of passenger limb movements that are characteristic of roller coaster experiences [6]. To address this gap, this study designs a non-standard safety envelope tailored for roller coasters by accounting for passenger limb movements, strictly adhering to EN 13814 and ASTM F2291:21 standards, fundamentally differing from the fixed geometric shapes (cylindrical and rectangular) used in industrial contexts. Inadequate safety envelopes can lead to collisions, accounting for 10% of roller coaster accidents globally between June 2017 and May 2018, underscoring the urgency to optimize their design [7]. The standard ASTM 2291:21 adopts a 95th percentile male anthropometric model to ascertain the maximum reachable space. This maximum reachable space is then expanded by 76 mm (3 inches) to define a safety envelope, ensuring adequate clearance for operational safety. This approach enables designers to customize the envelope dimensions based on risk assessments [8]. Meanwhile, EN 13814-1 and ISO 17842-1 specify empirical safety distance values for lateral, vertical, and leg dimensions, which are applicable to most roller coasters in operation. This standard empowers designers to construct a standardized safety envelope by selecting appropriate dimensions for vertical, leg, and lateral safety distances [6,9]. The current approach to measuring safety distances involves fabricating safety envelope frames from materials such as wood or PVC. These frames are mounted on roller coasters for visual inspection during operation. The method of drawing safety envelopes as outlined in the standard ASTM 2291:21 may result in irregularly shaped safety envelopes, which is highly subjective and not conducive to standardized measurement. The current manual inspection is prone to oversight in complex settings due to intricate tracks, large areas, and high speeds. EN 13814-1 and ISO 17842-1 enhance the reliability of safety envelope detection. This study utilizes these EN and ISO standards to define the safety envelope through lateral, vertical, and leg safety distances. This approach serves as the foundation for establishing a standardized safety envelope and assessing whether obstacles intrude upon it.

Furthermore, drawing on the experience of mobile entities using LiDAR for spatial detection [10,11] and moving object detection [12], this study applies LiDAR for distance measurement in the roller coaster industry. LiDAR sensors measure the distance by emitting a laser pulse and computing the laser time of flight. This active and direct ranging mechanism ensures accurate and efficient distance measurements. It achieves centimeter-level precision without additional triangulation and is unaffected by varying lighting conditions [13,14]. The rotating laser emitters within LiDAR facilitate comprehensive 360° scanning, ensuring that the safety envelope, which is also a plane, is inspected for intrusions without any angular blind spots. However, after detecting obstacles, accurately locating their positions in the roller coasters’ operational space remains challenging due to the complex layout of large-scale tracks. Traditional safety envelope frameworks require inspectors to visually monitor multiple obstacles to check for encroachments. With multiple potential risks along the track, this process often demands repeated trials to confirm adequate safety distances across all areas, making it time-consuming and requiring teamwork. While cameras enhance inspectors’ awareness of the surrounding environment, field experiments have shown that cameras have a limited field of view. The homogeneous scenery (e.g., green vegetation) beside tracks makes cameras only a supplementary tool to increase inspectors’ perception, and they are not capable of accurately pinpointing locations within the track area. If there were a positioning method that could precisely locate where an obstacle has intruded, it would greatly reduce the time and labor needed for inspections. The common positioning technologies, such as RTK (Real-Time Kinematic), UWB (Ultra-Wideband), and IMU (Inertial Measurement Unit), along with Kalman filter fusion applications [15], all have limitations. RTK suffers from signal stability issues during the roller coaster vehicle rotation. UWB and Wi-Fi have insufficient range for large roller coasters [16,17]. IMU-accumulated errors resulted in low positioning accuracy [18]. Due to the inaccuracy of the positioning sensor data, the data fusion results are also unable to meet the requirements. Considering the unique characteristics of roller coasters, which operate along fixed tracks with predetermined spatial coordinates [19,20], given these attributes, the DTW algorithm has attracted significant attention. Initially developed for isolated word recognition, pronunciation, and intonation evaluation in speech recognition [21,22,23], DTW has since become a versatile instrument applied across various scientific fields, including biology, economics, signal processing, robotics, and fault detection [24,25]. This algorithm, which aligns two signals by warping time, will now be employed in roller coaster track positioning. By synchronizing measured accelerations with the LiDAR point cloud, it can accurately pinpoint obstacles within the point cloud.

This study focuses on developing a novel safety envelope detector specifically designed for roller coasters. This innovative instrument offers the capability to customize the dimensions of the safety envelope, thereby catering to diverse operational requirements. Furthermore, it employs geometric methods to ascertain the presence of obstacles within the designated safety envelope. Additionally, the integration of an accelerometer and the DTW algorithm enhances obstacle positioning, ensuring a high degree of accuracy in locating potential hazards. Meanwhile, a camera is also used in conjunction with LiDAR, which captures images of the surroundings and enhances perception of the surrounding environment. This device is engineered to streamline the previously intricate process of safety distance measurement, offering an efficient and precise methodology that surpasses conventional approaches. Ultimately, the field testing has validated the practicality and efficacy of this instrument, demonstrating its ability to meet the demanding needs of the roller coaster industry.

## 2. Safety Envelope Measurement

**Figure 1:**
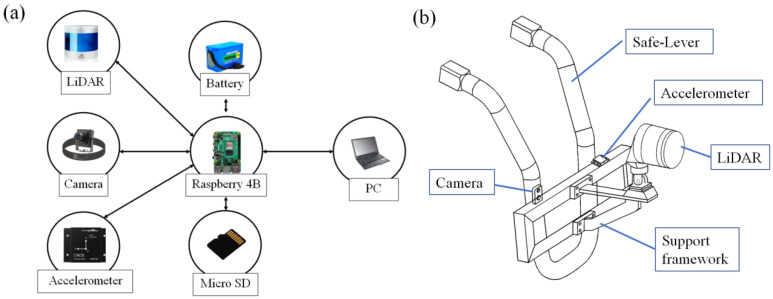
(**a**) SE-detector hardware architecture diagram; (**b**) SE-detector installation layout diagram.

The hardware of the SE-detector includes a PC, a Raspberry Pi 4 Computer, a 32-wire ouster LiDAR, a 64 GB micro-SD memory card, a 12 V 8000 mah battery, a 1080p wide-angle camera, and a three-axis accelerometer, as depicted in Figure 1a. The SE-detector is installed at a support framework in front of the vehicle, and the LiDAR extends forward to avoid being obstructed by other parts of the vehicle, as shown in Figure 1b.

**Figure 2:**
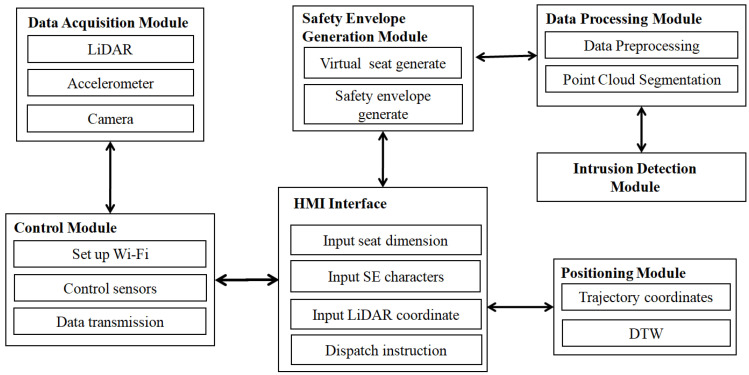
SE-detector software architecture and module functionality.

Figure 2 shows that the SE-detector includes data acquisition, a control module, a human–machine interface (HMI), safety envelope generation, data processing, intrusion detection module and positioning modules.

The SE-detector, mounted on the vehicle, collects point cloud, acceleration, and video data via its data acquisition unit. To adapt to the detection of various roller coasters, it is fixed on the safety bar using a self-designed support framework, as shown in Figure 1b, since nearly all roller coasters are equipped with Safe-Lever. The control module links the HMI software (V2.1), allowing it to send the initiation, cessation, and other commands to the data acquisition module. Using HMI-provided parameters, the safety envelope generation module designs the safety envelopes. Subsequently, the data processing module checks the point cloud data against safety envelopes for any violations, and the positioning module identifies the precise location of any obstacles along the track.

### 2.1. Data Acquisition Module

The data acquisition module includes a LiDAR, a three-axis accelerometer, and a camera, as depicted in Figure 1 and Figure 2. The LiDAR (Leishen Intelligent System Co., Ltd., Shenzhen, China) used in this paper has 32 channels, sends three point cloud datasets per second, and has a maximum range of 200 m. It can measure the distance of obstacles within a 360° coverage area around it, which is critical for obstacle detection around moving vehicles. By actively sensing depth through laser pulses, it achieves stable distance measurements (unaffected by ambient light), eliminating calibration needs and requiring minimal installation precision. In contrast, vision-based systems have a limited field of view and demand high installation accuracy, which is often impractical in environments like roller coaster maintenance. These advantages make LiDAR the dominant choice for such applications. A comparison of sensor advantages and disadvantages is presented in Table 1.

Accelerometers (Ruifen Technology Co., Ltd., Shenzhen, China) can measure the magnitude of acceleration in three mutually perpendicular directions. According to the experiment, the acceleration data in the direction of the human spine shows the best alignment result in the subsequent positioning algorithm. Therefore, this acceleration data is chosen for the subsequent positioning operations. The sampling frequency of the accelerometer is set to 100 Hz.

The camera is equipped with a Full High-Definition (FHD) sensor, offering a resolution of 1920 pixels by 1080 pixels, and is capable of capturing images at a frame rate of 30 frames per second (30 fps). And, it captures video images within a 120° range ahead. The LiDAR connects to the control module using UDP, while both the accelerometer and the camera use USB connections. To ensure synchronization across datasets from the LiDAR, camera, and accelerometers, which have different data collection rates, each dataset is timestamped by the control module. This practice is essential for subsequent data analysis and alignment.

### 2.2. Control Module

**Figure 3:**
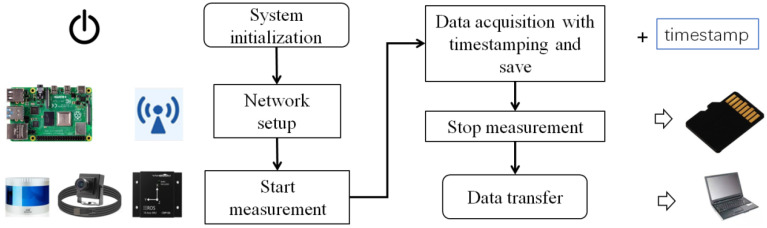
Control module flowchart.

The control module, as illustrated in Figure 3, is specifically designed to operate on a Raspberry Pi within an embedded Linux platform. This module is primarily responsible for directing the data acquisition process and ensuring accurate timestamping of each data packet. Further data processing and analysis are conducted by the other modules on the PC. Following its initialization, the control module sets up a Wi-Fi network to establish communication with the human–machine interface (HMI) module. Once this connection is established, the control module is ready to initiate detection upon receiving a start command. During the measurement process, the LiDAR, accelerometer, and camera will initiate operations, actively collecting data. The control module will append timestamps to the collected datasets and store them on a high-speed SD card, ensuring efficient data recording and synchronization. Upon receiving a data transmission command from the HMI, the control module transfers the point cloud, accelerometer, and video data back to the PC, ensuring that the data is readily accessible for further use.

### 2.3. Safety Envelope Generation Module

**Figure 4:**
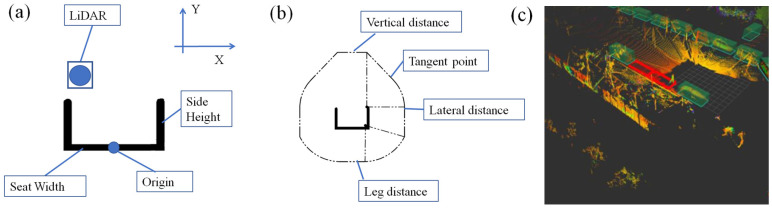
Safety envelope generation of (**a**) virtual seat and (**b**) safety envelope. (**c**) The safety envelope of cars [10].

The safety envelope generation module operates by initially setting the ‘Origin’ point, which is the middle of the virtual seat’s sitting surface, as depicted in Figure 4a. The dimensions of the virtual seat, LiDAR’s position, and safety envelope characteristics can be entered via the HMI. The term “seat width” denotes the width of the seat’s sitting surface, and “side height” indicates the height of the seat’s side. The LiDAR coordinates refer to the x and y coordinates of the LiDAR with respect to the ‘Origin’. The safety envelope characteristics refer to the vertical, lateral, and leg safety distances.

The virtual seat can be created through origin, seat width, and side height. And the safety envelope is created around the virtual seat, with outlines drawn based on the vertical, lateral, and leg safety distances. Figure 4b shows that the vertical and leg safety distance boundaries are parallel to the seat’s sitting surface, and the heights of these boundaries correspond to their respective safety distances. The lateral safety distance commences from the interior of the seat side, ascending in an arc and descending vertically. The endpoint of the vertical safety distance is connected to the lateral arc with a tangent. Subsequently, an arc is drawn using the corner of the seat’s sitting surface as the center, with the leg safety distance serving as the radius. This arc intersects the lateral perpendicular line, forming a comprehensive safety envelope. In contrast to the cylindrical or cubic shapes commonly used in other industries, as depicted in Figure 4c, this method of drawing safety envelopes fully considers the motion range of passengers’ arms and legs and also meets the requirements of roller coaster industry standards [6,9].

### 2.4. HMI Module

**Figure 5:**
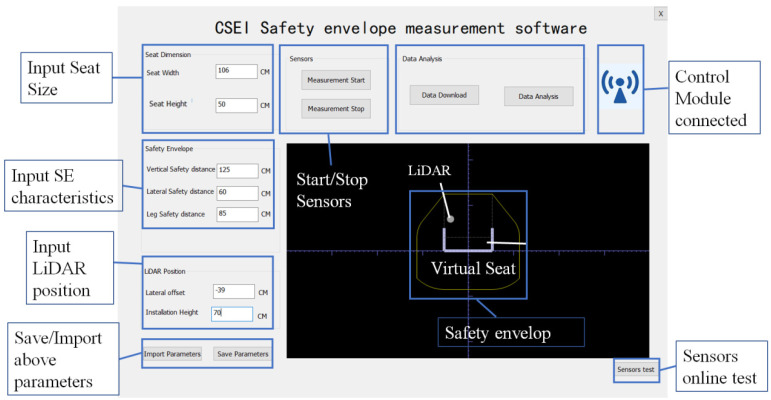
Functional overview of HMI software for safety envelope measurement.

The HMI module can communicate with the control module through the Raspberry Pi’s Wi-Fi. Users can input seat size, safety envelope characteristics, and LiDAR position here. As shown in Figure 5, the display area at the bottom right illustrates a schematic of the virtual safety seat, LiDAR, and safety envelope. This schematic is generated based on user-inputted parameters and is intended for adjustment purposes.

The HMI module sends start/stop commands to the control module, enabling synchronized sensor operation. Once detection is complete, data is transmitted to the PC for analysis via a download function. The HMI module features a data analysis button, which launches the data processing interface for the upcoming analysis of obstacle intrusions and positioning tasks.

### 2.5. Data Preprocessing and Point Cloud Segmentation

To enhance the accuracy of obstacle intrusion detection, this study designs a systematic data preprocessing pipeline. First, spatial range filtering is applied to the raw point cloud based on the effective scope of the safety envelope region, removing points beyond 6 m from the LiDAR scan center (such points lie outside the safety boundary and pose no intrusion risk). Second, statistical filtering is employed to eliminate outlier noise points, effectively suppressing noise interference. Notably, LiDAR-scanned point clouds exhibit a distinct near-dense and far-sparse distribution (with higher density in the near field and lower density in the far field). To reduce data volume while preserving the geometric features of obstacles, voxel grid downsampling is adopted in the downsampling stage. By setting a voxel size of 1 cm × 1 cm × 1 cm, this method reduces point cloud density without compromising obstacle shapes, significantly improving the computational efficiency of subsequent steps.

To address interference from vehicle point clouds (acquired simultaneously by the LiDAR during scanning) in obstacle detection, this study leverages the PointNet++ part segmentation network with multi-scale grouping (pointnet2_part_seg_msg) for point cloud segmentation. Specifically, 400 labeled point cloud samples are categorized into one semantic class (seg_classes) and two part categories (part categories), where the vehicle body is labeled as 0 and obstacles are labeled as 1. The model generates multiple local neighborhoods around each center point by simultaneously using varying neighborhood radii (e.g., r1, r2, …, rkk) and different numbers of K-nearest neighbors (e.g., K1, K2, …, Kkk). Features from these multi-scale neighborhoods are extracted and fused, enabling a comprehensive representation of both fine details (e.g., part edges) and global structures (e.g., overall part contours). Experimental results show that the model achieves up to 90% recognition accuracy for seat components. This not only helps specifically remove vehicle point clouds in later point cloud analysis stages but also effectively lowers false alarm rates.

### 2.6. Intrusion Detection Module

The data processing module is used to verify whether the point cloud measured by LiDAR is within the safety envelope. The point cloud data is represented by a spatial coordinate **P** (*X*, *Y*, *Z*) with the location of the LiDAR as the origin. But the safety envelope is defined with the middle of the seat’s sitting surface as the reference origin. A coordinate transformation is required for the point cloud data before it can be compared with the envelope boundary. It can be calculated by

(1) $$C^{\prime }=O+C.$$

where *C*′, *O*, and *C* are converted point cloud coordinates, coordinates of LiDAR, and coordinates of the point cloud, respectively.

**Figure 6:**
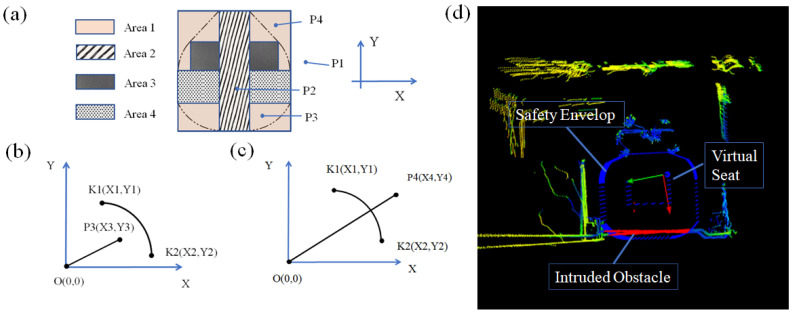
Point cloud classification algorithm based on spatial regions: (**a**) classification of point clouds **P1**–**P4** in predefined regular areas (Area 1–Area 4), (**b**) classification of point clouds within irregularly shaped areas (within safety envelope), (**c**) classification of point clouds within irregularly shaped areas (out of safety envelope), and (**d**) final classification results with safety envelope, virtual seat, and intruded obstacle annotations.

This study employs geometric methods to determine whether point **P** (*X*, *Y*, *Z*) intrudes the safety envelope, where *X*, *Y*, and *Z* denote lateral, vertical, and motion-direction coordinates, respectively. As the safety envelope is defined on the cross-sectional plane orthogonal to the motion direction (*Z*-axis), the *Z*-coordinate is irrelevant to intrusion detection. The judgment is initiated by verifying if **Ps** (*X*, *Y*) coordinates fall within Area 1 (the rectangular bounding box of the envelope) or its sub-regions (Areas 2–4), as visually decomposed in Figure 6a. Points outside Area 1 (e.g., P1) are directly excluded. Points inside Area 1 undergo secondary checks: those within Areas 2–4 (e.g., **P2**) are confirmed as intrusions, while others (e.g., **P3** and **P4**) require further geometric analysis via line–segment intersection (Figure 6b,c). For points like **P3** and **P4**, we construct line segment *O*-*P* (*O*: seat origin in Figure 4a) and apply geometric intersection detection with envelope boundaries. This process is accelerated using the Shapely library for efficient line–polygon collision checks.

## 3. Positioning Algorithm

Detecting and localizing obstacles that intrude into the safety envelope presents a challenge, due to roller coasters’ complex layout and rapid movement. Traditionally, inspectors had to monitor areas prone to insufficient safety distances, checking for interference between the vehicle’s attached safety frame and obstacles in risk zones. This process often involved multiple personnel or repeated observations. The camera can only serve as an auxiliary means to enhance the inspectors’ perception of the scene, and it cannot accurately determine the location of obstacles on the roller coaster. To address this issue, this paper presents an accelerometer-fused DTW positioning algorithm for precise obstacle localization. DTW outperforms alternatives (RTK, UWB, and visual SLAM) by eliminating infrastructure dependencies and maintaining robustness under dynamic occlusions or environmental disturbances (e.g., lighting changes and rain/fog), ensuring continuous localization during high-speed operation.

### 3.1. Basic Theory

The DTW algorithm aligns two discrete time series by warping their time axes to find an optimal one-to-one matching between their points. For the discrete time series **A**[*n*] and **B**[*m*], the matching can be represented by a path **W** = {**W_1_**, **W_2_**, …, **W_n_**}, where **W_k_** = (*i*, *j*), *i* ∈ [1, *n*], and j ∈ [1, *m*], indicating a_i_ and b_j_ of **A**[*n*] and **B**[*m*].

**Figure 7:**
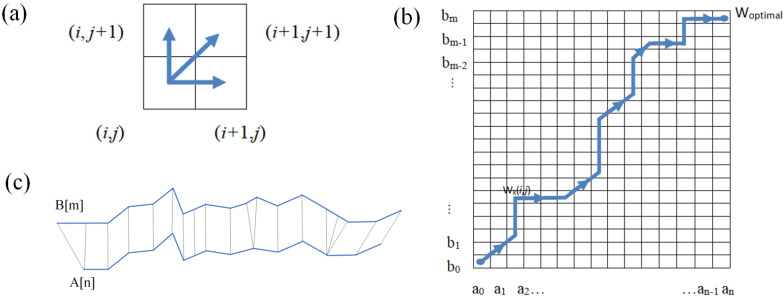
Illustrations of key elements in the DTW algorithm: (**a**) forward path; (**b**) minimum total cumulative distance; and (**c**) best match via optimal path.

The path must begin at (1, 1) and end at (*n*, *m*). The path progression is constrained: if **W_k_** = (*i*, *j*), the next point **W_k+1_** can only be (*i* + 1, j), (*i* + 1, *j* + 1), or (*i*, *j* + 1), as illustrated in Figure 7a. The local distance between aᵢ and bⱼ is defined as *Dist*(*i*, *j*) *=* (*a_i_ − b_j_*)^2^. The goal is to find the path that minimizes the total cumulative distance. This is achieved using a cumulative distance matrix **D**[*n*, *m*], which is computed in a forward manner through dynamic programming:

(2) $$D\left(i,\;j\right)=Dist\left(i,\;j\right)+\left\{\begin{array}{c} D\left(i-1,\;j\right)\\ D\left(i,\;j-1\right)\\ D\left(i-1,\;j-1\right) \end{array}\right.$$

The endpoint element **D**(*n*, *m*) of the cumulative distance matrix represents the total cumulative distance of the optimal paths for sequences **A**[*n*] and **B**[m], which can indicate the similarity between the two sequences. The best match between **A**[*n*] and **B**[m] according to the optimal way **W_optimal_** is depicted in Figure 7b. The smaller the **D**(*n*, *m*), the better the matching degree between the two sequences. Based on the optimal alignment path **W_optimal_**, the best correspondence between the two sequences can be identified, as illustrated in Figure 7c.

### 3.2. Algorithm Application

**Figure 8:**
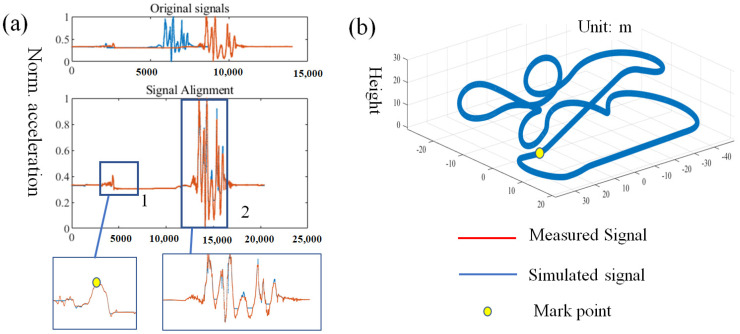
Obstacle positioning process: (**a**) alignment of measured and simulated signals; (**b**) mark point in roller coaster’s layout.

The accelerometer equipped with this device will measure the acceleration of the roller coaster, and the simulated acceleration can also be provided by the design company. Despite the apparent similarities between the measured and simulated acceleration profiles, intrinsic differences exist due to the complex forces encountered during the vehicle operation, such as friction and wind resistance. These factors introduce variations in the duration and amplitude of the acceleration sequences. However, the DTW algorithm essentially matches two sequences by allowing an element in one sequence to correspond to multiple elements in the other, thereby finding the minimum Euclidean distance between the sequences to achieve optimal alignment. As long as the sequences have distinct characteristics, minor variations in details do not affect the matching. Even when dealing with acceleration pairs of unequal lengths, the matching outcome remains almost unaffected, as demonstrated by the measured and simulated accelerations in ‘Original signals’ of Figure 8a. To achieve a good matching effect, this paper selects the vertical acceleration data, which is the acceleration parallel to the direction of the passenger’s spine, for comparison. The sampling frequency for both the measured acceleration signals and the simulated acceleration signals is 100 Hz. To mitigate the effects of noise, the measured data was subjected to 1 Hz Butterworth low-pass filtering, which was informed by experimental findings that this frequency provides optimal matching outcomes. Following filtering, normalization of both the simulated and measured time series is required before proceeding with the matching process.

In Figure 8a, for ‘Signal Alignment’, the DTW algorithm demonstrates optimal matching performance by twisting the originally shorter simulated data to match the longer measured data. This is particularly evident in parts 1 and 2, where the measured and simulated acceleration values are essentially perfectly matched. The precise alignment is crucial for the subsequent obstacle positioning.

To validate this positioning algorithm, a section with a distinct characteristic was selected from the actual measured acceleration, as indicated by the yellow dot in the detailed section 1 of Figure 8a. After positioning with the DTW algorithm, the corresponding location is marked in Figure 8b. This matches the actual situation because, at the start, the train moves forward slowly with minimal acceleration fluctuation. Then, as the train connects with the lifting hook in the lifting section and begins to be lifted, there is a brief change in acceleration. Subsequently, as the train body is slowly lifted by the lifting hook, the acceleration stabilizes again. This preliminary verification confirms the effectiveness and accuracy of the positioning algorithm. In the sections that follow, an obstacle will be employed to evaluate the equipment’s operational effectiveness and precision.

## 4. Application

**Figure 9:**
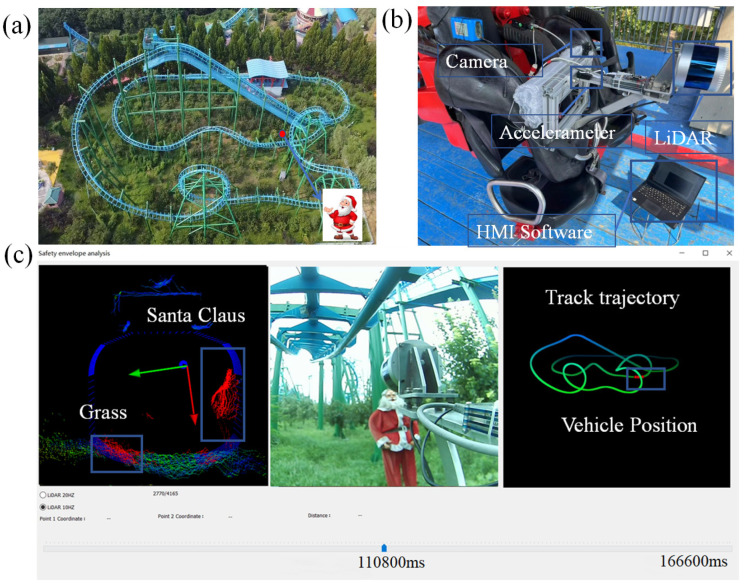
On-site test images: (**a**) test roller coaster photo, (**b**) SE-detector hardware installation photo, and (**c**) analysis software interface displaying a point cloud on the left panel, camera view in the central panel, and positioning map on the right panel.

The devised instrument was implemented to assess safety distances on a medium-sized roller coaster, as shown in Figure 9a, located in Xingtai, China. The selected roller coaster covers an area of 5700 square meters, with a track length of 500 m and a maximum operating speed of 74.4 km/h. It is designed with two seats per row and a maximum capacity of 14 passengers. The track features various structures, including lift hills, vertical loops, spirals, and saddle-shaped turns. The roller coaster’s vehicle is suspended beneath the track, allowing passengers the freedom to move their arms and legs. To enhance visual clarity, a Santa Claus figure was chosen as the obstacle. Although a smaller obstacle could still be detected, it would have produced a trajectory resembling only a line segment or polyline, which is less intuitive.

To prevent being blocked by the front seats, the SE-detector was specifically mounted in front of the safety lever on the first row of roller coaster seats, as shown in Figure 9b. The safety envelope encompasses an entire row of adjacent seats; thus, the specified seat width pertains to the collective width of a row, not an individual seat. According to the EN standards, based on the vehicle’s operating speed and the side height, the lateral safety distance, upper safety distance, legroom safety distance, and other parameters required for the HMI software are chosen as shown in Table 2 [6].

Before the inspection, the design organization provided the three-dimensional coordinates of the track and the simulated acceleration values. Specifically, a “Santa Claus” figurine, serving as a mock obstacle, was positioned within the coaster’s path.

After the detection, the SE-detector identifies the obstacle (Santa Claus), as depicted in Figure 9c. The point cloud data indicates Santa Claus in red because he has entered the designated envelope line, and his presence has been detected by the camera system. His position is also accurately pinpointed along the roller coaster track, which corresponds to the actual placement.

The minimum detectable object size depends on the field of view, scanning frequency, and distance. Under stationary conditions, the detection limits can be found in Table 3 below.

The false rate is primarily determined by point cloud classification accuracy. The current PointNet++ model achieves 90% classification accuracy but is limited by insufficient training sample diversity. Positioning accuracy depends on the accelerometer’s sampling frequency and vehicle speed. For most roller coasters (max speed: 20–30 m/s), a 100 Hz sampling rate ensures positioning errors ≤0.2~0.3 m, with higher accuracy at lower speeds.

## 5. Conclusions

In contrast to conventional PVC pipe or wood-woven frame-based methods, this study developed a novel safety envelope (SE) detector instrument for intrusion detection. The cost of the SE-detector was dominated by the 32-channel LiDAR module (90% of the total), whose price has significantly decreased. As the price decreases, the device becomes increasingly significant for promotion.

Compared to other visual detection methods, it offers the advantages of low installation precision requirements and a wide scanning field of view. By setting safety envelope parameters, it can meet various vehicle body requirements, and detection results are recordable. Technically, it complies with ASTM, EN, and ISO standards and qualifies for official recognition as a formal inspection method. Additionally, this paper innovatively applied the dynamic time warping (DTW) algorithm—originally used in speech recognition—to locate moving objects along fixed trajectories, which is a breakthrough finding. In comparison to positioning technologies such as RTK, IMU, and UWB, it exhibits immunity to signal transmission interference, independence from operational area size, and absence of cumulative error. Future research can continue to expand this direction for scenarios with similar needs.

SE-detectors may fail in specific scenarios due to two main issues: high-speed target detection and low point cloud segmentation accuracy. First, obstacles beside extremely fast-moving roller coasters may exit the LiDAR’s field of view because of the limited rotating speed of laser emitters. It can cause missed detections. Second, limited training data for point clouds leads to poor feature learning in complex environments (e.g., seats as interference), resulting in segmentation errors and false alarms. Currently, false alarms require manual verification. These challenges can be mitigated by adopting higher-speed LiDARs and improving segmentation models with expanded labeled datasets. This will be our future research direction.

## Figures and Tables

**Table 1:** Performance comparison of vision and LiDAR sensors [26].

Sensor	Advantages	Disadvantages
Monocular Camera	Low price, suitable for close-range measurement	Limited depth perception, sensitive to lighting conditions, limited field of view, susceptible to distortion
Stereo Camera	Strong depth perception, insensitive to lighting conditions, suitable for long-range measurement	Higher cost, requires calibration, susceptible to distortion, limited field of view, not suitable for transparent or reflective surfaces
LiDAR	High accuracy, suitable for outdoor environments, broad field of view, capable of working in low- or no-light conditions	Higher cost, larger size, limited resolution

**Table 2:** HMI module input characteristics.

Modules	Description of Characteristics		Values of Characteristics
Seat size		Seat width	106 cm
		Side height	56 cm
SE characteristics		Vertical safety distance	150 cm
		Lateral safety distance	100 cm
		Leg safety distance	100 cm
LiDAR position		Lateral offset	−39 cm
		Installation height	70 cm

**Table 3:** The minimum detectable object size.

	Scanning Frequency 10 Hz		Scanning Frequency 20 Hz	
Distance (m)	Longitudinal Resolution (cm)	Lateral Resolution (cm)	Longitudinal Resolution (cm)	Lateral Resolution (cm)
1	1.7	0.3	1.7	0.6
2	3.5	0.6	3.5	1.2
3	5.2	0.9	5.2	1.8
4	7.0	1.2	7.0	2.4

## Data Availability

The original contributions presented in this study are included in the article. Further inquiries can be directed to the corresponding authors.
